# The needs of families accompanying injured patients into the emergency department in a tertiary hospital in Gauteng

**DOI:** 10.4102/curationis.v39i1.1567

**Published:** 2016-06-24

**Authors:** Meghan L. Botes, Gayle Langley

**Affiliations:** 1 Department of Nursing Education, University of the Witwatersrand, South Africa

## Abstract

**Background:**

Families are not prepared for traumatic injuries of loved ones. Emergency nurses have the important role of caring for patients and families in this time of crisis. Family needs in the critical care setting have been explored using the Critical Care Family Needs Inventory (CCFNI), however little is known about family needs in the emergency department.

**Objectives:**

This study sought to determine the needs of family members accompanying injured patients into the emergency department, and if these needs were met.

**Methods:**

A quantitative, descriptive, study was conducted in a level 1 trauma facility in Johannesburg, South Africa. The population included families of patients admitted to the emergency department, sampling 100 participants. The instrument, based on the CCFNI, was validated in a pilot study in Melbourne, Australia and re-evaluated using the Cronbach Alpha validity test to ensure internal consistency.

Five themes were explored: ‘meaning’, ‘proximity’, ‘communication’, ‘comfort’ and ‘support’ and data were analysed using descriptive statistics. Responses to open-ended questions were analysed using content analysis. Permission from the Human Research Ethics Committee was granted and participants were ensured confidentiality and the option for counselling if required.

**Results:**

Themes ranked highly important were ‘meaning’ and ‘communication’. Satisfaction was highest for ‘meaning’. Low satisfaction levels for ‘communication’ were found. Issues regarding prolonged time spent in the emergency department and discrimination were raised.

**Conclusion:**

These findings have a negative impact on the family’s satisfaction with care and it is recommended that the nurse’s role in family care be further explored and emphasised.

## Introduction

Traumatic injuries have been described by the World Health Organization as a global health problem. They account for 5.8 million deaths per year, a devastation that surpasses that of other global epidemics such as malaria, tuberculosis and HIV (WHO 2010:3). Globally, the top three leading causes of death by injury result from road traffic crashes, homicide and suicide (WHO 2010:3). This tragic phenomenon affects high-income countries, and has an even greater impact on lower to middle income countries because of the scarcity of resources, as described by Chandran, Hyder and Peek-Asa (2010:110).

In South Africa, traumatic injuries fall within the top 10 leading causes of death nationwide (Norman *et al*. 2003:5). Nearly three-quarters of the South African population experience a traumatic event and just under half experience trauma to, or the unexpected death of a loved one (Williams *et al*. 2007:846). Hardcastle, Samuel and Muckart (2013:1550) have predicted that the incidence of trauma in national public hospitals in South Africa is approximately 750 000 cases per year.

Death and disability are not the only consequences of traumatic injuries. The emotional turmoil which is thrust upon a family in the event of sudden traumatic injury to a loved one can be debilitating and families are dependent on the health professionals in the emergency department not only to give acute care to their loved one but also to walk them through the process of dealing with the current crisis (WHO 2010:12).

The speciality field of critical care nursing has added knowledge and literature to the concept of family centred care (Linnarson, Bubini & Perseius 2009:3103). Research has defined the needs of families and advocated that the role of the nurse includes the care of the families of patients (Verhaeghe *et al*. 2005:507). The needs of family members of patients in a critical care setting have been identified and grouped into 5 main categories namely: ‘proximity’, ‘meaning’, ‘communication’, ‘support’ and ‘comfort’.^7^

The theme proximity takes into account the family’s access to their injured loved one, while comfort refers to the emotional and physical comfort offered by the staff and facilities available in the hospital. Support does not only refer to support offered by staff but also whether staff recognise and allow for the family to make use of their own social support structures. Communication does not only refer to transfer of information but encompasses the way in which information is shared, how regularly information is given and the how interactive the process of communication is. Meaning enables families to cope with the crisis at hand by ensuring that communication is honest while still offering hope (Linnarson, Bubini & Perseius 2009:3106).

The emergency department setting is fast paced and unpredictable in nature (Cardona *et al*. 1994). This places the family and the nurse in vulnerable positions. The emergency nurse needs to have a unique ability to deal with a variety of stressors simultaneously while caring for the patient and the family. Stressors within the emergency unit include violence, heavy workload and poor mix of skills within the emergency care team (Healy & Tyrell 2011:32); (Ross-Adjie, Leslie & Gillman 2007:121). In developing countries such as South Africa, the challenges include dealing with the entry of new patients while managing the existing admissions of seriously ill patients, with limited resources, few skilled nursing staff and a backlog of patient admissions (Wentzel & Brysiewicz 2014:95). Prioritising emergencies, while simultaneously dealing with the anxieties of the families of injured patients brought into the unit, is challenging (Wentzel & Brysiewicz 2014:95). This role can be difficult to navigate and fulfil in its entirety if the needs of the family in this setting are not identified and understood. Once the expectations of family members are defined, the role of the nurse can be built upon this understanding in order to address these needs effectively.

The needs of families in the critical care setting have been studied using the Critical Care Family Needs Inventory (CCFNI). However, few studies have been conducted in the trauma and emergency setting and it cannot be assumed that the needs of families are the same in both environments (Linnarsson, Bubini & Perseius 2009:3108; Redley & Beanland 2004:95). Therefore the purpose of this study was to determine the needs of families accompanying patients into the emergency department and to assess their level of satisfaction with the care received.

## Methods

A quantitative, descriptive, design study was conducted in a level 1 trauma care facility in a public tertiary academic hospital in Johannesburg, South Africa, where an average of 952 patients is seen per month as recorded in the hospital’s monthly statistics. This emergency department specialises in the acute care of patients with traumatic injuries.

The population comprised families of patients brought into the emergency department who were literate in English, over the age of 18, cognitively intact to participate in the study and were present in the hospital at the time of being approached to participate in the study. If at any time the researcher encountered a family with signs of emotional distress, the family was given the option to be referred to an advanced psychiatric nurse (who had given her assent), for further counselling. Family members included any next of kin or spokesperson elected by the family or patient. Participants were approached in the waiting area of the hospital either while waiting for files to be compiled or after having seen their injured relative. Families who appeared overly distressed were excluded from the study.

Permission from the Human Research Ethics Committee, from the relevant academic institution and all relevant authorities was sought and granted (Ethical Clearance certificate M130133). Participants were given a letter of information and the posting of an anonymous completed questionnaire into a sealed box was taken to imply consent.

A power analysis was completed with the *p*-value set at 0.1 and effect size of 95%, which calculated a sample size of 97 participants. One family member accompanying the injured patient was asked to participate. Using convenience sampling, the sample consisted of two groups of participants. A minimum of 50 participants were approached upon entering the emergency department to complete the first questionnaire (CCFNI-1) as participants at this stage would not yet have experienced the services rendered by the emergency department staff and would not be influenced in their answers. The second group of 50 different participants accompanying different patients was approached upon leaving the emergency department to complete the second questionnaire (CCFNI-2), having had enough experience in the emergency department to determine whether their needs were met. The two groups were not made up of the same people as time spent in casualty varies from one patient to the next and follow-up on the same family members would be very challenging. It also allowed for family members who have been too emotional to opt out after completing the first questionnaire.

An instrument was formulated to determine the needs of families accompanying patients into the emergency department (Redley *et al*. 2003:609). By adapting the CCFNI for the emergency setting, an instrument consisting of needs statements rated according to importance (CCFNI-1) and a second instrument ranking needs statements according to the level of satisfaction (CCFNI-2) was compiled (Redley *et al*. 2003:609). The instrument made use of the Likert scale format and addressed five principle needs, namely ‘meaning’, ‘proximity’, ‘communication’, ‘comfort’ and ‘support’. Open-ended questions were included at the end of the instruments, which asked participants about helpful or unhelpful behaviours of staff in the emergency department and required general comments.

## Reliability and validity

The original instrument was tested during a pilot study in Melbourne, Australia. An inter-rater agreement level of 90% was determined to ensure relevance of the items and to ensure reliability and was later tested in a pilot study conducted in an emergency department in Melbourne (Redley *et al*. 2003:609). For the purpose of this study, the instrument was adapted for the South African context in terms of language and wording, using colloquial substitutions for certain terms. The instrument was then re-evaluated using the Cronbach Alpha test to ensure internal consistency at a subscale level for items within each domain. An alpha value ranging between 0.70 and 0.95 was considered acceptable. If alpha values for a domain were below or above this range, justification for use of this data was provided (see Table 1).

**TABLE 1:** Results Cronbach alpha test.

Variable	Theme	Alpha value
**CCFNI-1**	Meaning	0.7457
	Proximity	0.7686
	Communication	0.6483
	Comfort	0.7246
	Support	0.6811
**CCFNI-2**	Meaning	0.9262
	Proximity	0.8502
	Communication	0.903
	Comfort	0.8515
	Support	0.8812

CCFNI, Critical Care Family Needs Inventory.

*Source*: Authors’ own work

Within the domain ‘communication’, three items were found to weaken the Alpha value, and within ‘support’ four items were reviewed. The difference in Alpha values upon removal of these items was miniscule. The items were relevant to the domain and therefore included.

Descriptive statistical analysis was used to explore the collected data. Data were captured on a Microsoft Excel spread sheet and then cleaned and coded. The data were then imported to STATA version 12.0 statistical software for analysis and organised into the five major categories which the instrument addresses. Using a Likert scale, scores were allocated to the items within the categories to determine values. Content analysis was used for analysis of responses to the open-ended questions. Responses to the open-ended questions were recorded and analysed. Analysis of responses included identifying existing and recurring concepts and grouping these concepts into categories. Eight categories were identified: communication, proximity, time taken to be attended to, friendliness or caring gestures, professionalism, treatment of the patient, equality and physical needs of family members.

## Results

In total, 50 questionnaires for CCFNI-1 and 50 questionnaires for CCFNI-2 were received between May and June 2013. Participants who answered the first questionnaire (CCFNI-1), consisted of 53% (*n* = 26) female participants and 47% (*n* = 24) male participants. The mean age of participants was 38.2 years of whom 40% (*n* = 20) fell within the age range of 25–36. The relationship of the participants to the patients varied in that 12% (*n* = 6) were married spouses of the patient, 19% (*n* = 10) were unmarried partners, 14% (*n* = 7) were parents of the patients, 16% (*n* = 8) were siblings of the patients, 18% (*n* = 9) were children of the patients and 21% (*n* = 10) other, which included friends, neighbours and colleagues. One participant did not respond to this question.

Participants who completed the second questionnaire (CCFNI-2) comprised 61% (*n* = 28) females, with a mean age of 38.2 with 40% (*n* = 20) falling within the age range of 25–36. The relationship of the participants to the patients varied in that 25% (*n* = 12) were married spouses of the patient, 6% (*n* = 3) were unmarried partners, 14% (*n* = 7) were parents of the patients, 33% (*n* = 16) were siblings to the patients, 6% (*n* = 3) were children of the patients and 14% (*n* = 7) other, which included friends, neighbours and colleagues.

**FIGURE 1:**
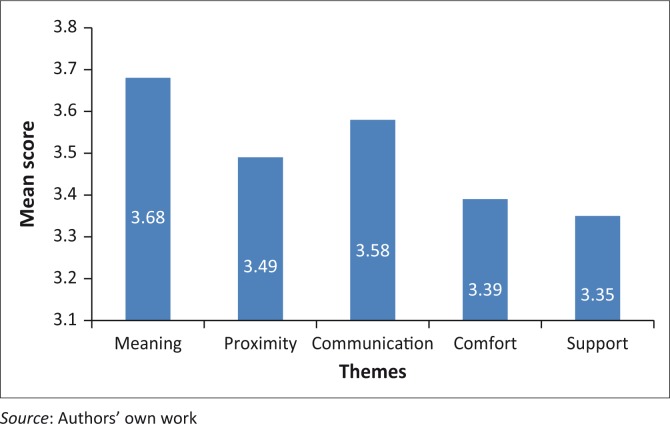
Overview of results for themes of CCFNI-1 (level of importance).

**FIGURE 2:**
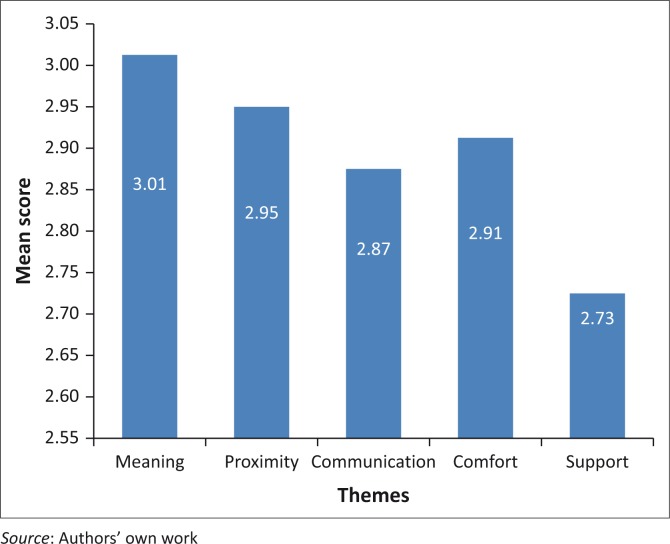
Overview of results for themes of CCFNI-2 (level of satisfaction of needs met).

Needs statements within the instrument were assigned to one of the five themes; scores were allocated by assigning values to the Likert scale, with 1 indicating the lowest score and 4 being the highest. Means for each statement individually as well as for each theme were calculated and analysed accordingly. Results from CCFNI-1 reported on what families ranked as being most important while the results of CCFNI-2 reported on their level satisfaction with the services for meeting their needs (see Figure 1 and 2).

The theme ranked with the highest mean score for identified needs was ‘meaning’ and the lowest was ‘support’ (see Table 2).

**TABLE 2:** Top 10 ranked needs statements CCFNI-1 (level of importance).

Ranking	Question	Statement	Total score	Mean score	Mode	Theme
1	17	To be assured that the best care possible has been given to your relative	197	3.86	4	Meaning
2	30	To feel hospital staff care about your relative	188	3.84	4	Meaning
3	15	To have questions about the condition of your relative answered honestly	195	3.82	4	Meaning
4	12	To talk to a nurse	191	3.82	4	Communication
5	6	To have explanations given in understandable terms	193	3.78	4	Communication
6	7	To be kept updated frequently	193	3.78	4	Communication
7	14	To know about the expected outcome of your relative	188	3.76	4	Meaning
8	35	To feel like there is hope	192	3.76	4	Meaning
9	11	To talk to a doctor	192	3.76	4	Communication
10	28	To feel accepted by hospital staff	188	3.76	4	Comfort

CCFNI, Critical Care Family Needs Inventory.

*Source*: Authors own work

The top 10 ranking statements were analysed according to mean scores, 5 of the top 10 ranking statements belonged to the theme ‘meaning’, while 4 belonged to the ‘communication’ theme and 1 belonged to the ‘comfort’ theme.

The theme with the highest mean score was found to be ‘meaning’. The lowest scoring theme was ‘support’ (see Table 3).

**TABLE 3:** Top 10 ranked needs statements CCFNI-2 (level of satisfaction).

Ranking	Question	Statement	Total score	Mean score	Mode	Theme
1	35	To feel like there is hope	153	3.40	4	Meaning
2	39	To have toilet facilities nearby	147	3.34	4	Comfort
3	30	To feel hospital staff care about your relative	-153	3.33	4	Meaning
4	17	To be assured that the best care possible has been given to your relative	149	3.24	4	Meaning
5	31	To be assured of the comfort of your relative	135	3.21	4	Comfort
6	19	To see your relative as soon as possible	143	3.18	4	Proximity
7	28	To feel accepted by hospital staff	134	3.12	4	Comfort
8	1	Have a doctor or nurse meet you on arrival at the hospital	151	3.08	3 & 4	Support
9	29	To be treated as an individual	129	3.07	4	Meaning
10	27	To have time alone with your relative	135	3.07	4	Proximity

CCFNI, Critical Care Family Needs Inventory.

*Source*: Authors own work

The highest ranking needs statement with regards to satisfaction of needs met was 35. ‘To feel like there is hope’. Needs statements ranked in the top 10 were derived from the themes meaning’, ‘comfort’, proximity’ and ‘support’. No needs statements from the theme ‘communication’ were included in the top 10 ranked items.

## Responses to open-ended questions

Categories emerging from the data included communication, proximity, time taken to be attended to, friendliness and gestures of caring, professionalism, treatment of the patient, equality and physical needs of the participants. Responses from both groups of participants were similar and categories were identical. Of all the categories, communication, time taken to be attended to and equality were met with the most dissatisfaction with mostly negative responses regarding these issues. Participants verbalised frustrations about poor communication and prolonged waiting times and references were made to discrimination based on nationality. Responses related to communication included: *nothing was explained to us and scan results took eight and a half hours to come, was not told the results.* Family members also voiced their frustration with not being allowed to stay with their injured relative: *My son is injured and I’m not allowed to be with him during trauma, it was not busy at the casualty but we were told to sit outside.* Families also expressed their perception of discrimination in responses such as the following: *being told by the administration officers that I am a foreigner of which I know and I don’t want anyone to remind me of that, it feels like foreigners are less human beings*.

Some participants however, remarked positively about the emergency department staff and described them as being friendly and professional: *They were all helpful and very much caring.*

## Discussion

Needs related to ‘meaning’ were ranked as most important. The need to derive meaning from the crisis situation that families find themselves in when a relative is injured has been highlighted as a means of developing an ability to cope with the anxiety and stress of the crisis (Redley, Beanland & Botti 2003:94). Highly ranked needs in this theme included the need for honest information, as well as the need to feel that there is hope and to be assured of the best care for their injured relative. Traumatic injuries in South Africa are often related to violence (Norman *et al*. 2007:653); and this sudden calamity can produce much anxiety, which can be reduced by the care of the emergency department’s staff members. The needs that were met with the greatest satisfaction in this study belonged to the theme ‘meaning’ which is a positive finding. The depth and quality of the therapeutic relationship between the nurse and the family members of the patient forms an important part of the family’s experience in the emergency department and their ability to deal with the prognosis of their loved ones.

The second most important theme ranked by families was ‘communication’, and unique to this study was the ranking of the need to talk to a nurse. Families ranked this as the most important communication need, above the need to talk to a doctor. In the ‘communication’ theme, families expressed the need to talk to a nurse, to be given explanations in understandable terms and to be kept updated frequently, as highly important needs. The anxiety of not knowing about the condition of an injured loved one can be reduced by ensuring good communication between the nurse and family (Linnarsson *et al*. 2009:3106). The results of this questionnaire (CCFNI-2) indicated a lack of satisfaction with communication-related aspects. Families expressed their dissatisfaction with lower mean scores in needs statements, as well as in the open-ended questions. A pilot study was conducted in an emergency department in Melbourne, following the development of the tool adapted for this setting. The findings were different as the communication of nurses was a well met need with greater satisfaction in the Australian study (Redley *et al*. 2003:613). This raises questions about the difference in families’ satisfaction with communication and might indicate an area for further research in South Africa.

The theme ranked as being the next most important need was that of ‘proximity’. Families desire to be able to see their injured relatives. The emergency department used for the purpose of this study made use of an open visiting policy with no fixed visiting times. However families were restricted to short visits at a time. Research has shown that units with open visiting policies derived a higher level of satisfaction of families with regard to proximity (Cook 2006). ‘Proximity’ was ranked as being second most important among the five themes in terms of satisfaction of needs met. Families were satisfied with their accessibility to their injured relative.

Needs related to ‘comfort’ and ‘support’ were not ranked as being highly important. However, to feel accepted by hospital staff was included in the top 10 most important needs statements. Emotional comfort is a means of reassuring the families about their importance and identity (Redley *et al*. 2003:608); this acknowledgement leads to a greater level of trust and satisfaction (Linnarsson *et al*. 2009:3107).

Data, emerging from responses to the open-ended questions, were largely supportive of the findings in the questionnaire. However, from the categories that emerged from the open-ended questions of both questionnaires, two were highlighted as being important for future research and for the needs of families accompanying injured relatives into the emergency department. Time spent in the emergency department was prolonged according to the responses of the study’s participants. This has a considerable influence on the level of satisfaction of care, as the longer an injured relative is kept in the emergency department; the more negative the experience becomes (Taylor & Benger 2004:530). Time spent in the emergency department also has a considerable influence on the comfort of the family as waiting areas are not well equipped and anxiety is heightened when away from the injured relative (Karlsson *et al*. 2011:15). Facilities should cater for the waiting family’s needs and effective communication and increased proximity might reduce this anxiety.

Families desire to be treated equally, regardless of race and nationality. This is of particular importance in a South African context, where history has shown issues of equality to be a source of conflict and current incidents indicate that xenophobia is on the rise (Landau 2011). This expressed need for equality may be attributed to the surrounding area of the hospital which is urban and densely populated with locals and foreigners. Xenophobia is not limited to a South African context. Globally, migration is often perceived as a threat to the local society, and as a result, xenophobia is expressed in both subtle and more explicit ways. Developing countries are a popular destination for migrants from other high income countries as well as lower to middle income countries and resentment may ensue as a result (Crush 2009). Unfortunately, the anti-immigrant sentiment of a country has the potential to infiltrate the health care system with foreign patients on the receiving end of discriminatory care (Achiume 2014:332).

An encouraging finding of this study was the positive remarks about equality of care with regard to race. Families in this setting identified this as an important need, and were satisfied with the equality of care received based on race. This is an affirmation of the growth of post-apartheid South Africa. However it does not fully address the need for equality related to nationality. Nurses need to become aware of their own personal difficulties related to these issues, as well as the implications for patients and their families. Nurses’ own beliefs have been identified as a hindrance to embracing family-centred care (Saveman 2010:36). Many tertiary training institutions in South Africa oblige graduating nurses to swear to the ‘Hippocratic Oath’ as well as the ‘Nurse’s Pledge of Service’. Both of these oaths bind the nurse to a commitment to care for patients regardless of race or nationality.

## Limitations

These findings are not necessarily applicable to the total population of South Africa and cannot be generalised. A lower internal consistency score on two of the domains of the questionnaire also decreased the validity of the questionnaire. The questionnaire did not include demographics such as education, culture, nationality and preferred language which may have influenced the findings. By excluding people who could not communicate in English the sample was limited. The use of two separate cohorts for each questionnaire could limit the ability to generalise that the needs of both cohorts were the same. No interviews were conducted with health care providers. Consequently their experiences and perceptions remain unknown.

### Implications for emergency nurses

The findings of this study have important implications for the clinical practice of the emergency nurse. From its history, trauma and emergency nursing has had as its focus, the patient in the emergency room. This focus is evolving and needs to include the important aspect of caring for families of patients admitted into the emergency department. The role of the nurse in communication has been emphasised and needs to be realised in practice. Families have expressed their need for support and communication from the nurse, who in turn may offer more holistic nursing practice, taking into consideration the family in crisis. The burden of crime and traumatic injuries in South Africa is considerable and emergency nurses may become overwhelmed by the influx of patients. It is important that consideration for the needs of the family become a part of the emergency nurse’s role and responsibility.

Issues of discrimination based on race and nationality are of great concern in post-apartheid South Africa and the equality of patients should be reflected in the care of all professional nurses.

## Recommendations

There is a need for the role of the emergency nurse with regard to family care to be further explored. Further research may define this role more practically and suggest methods of meeting the needs of family members while dealing with the stressors of multiple responsibilities within the emergency department. Research should include the perspectives, needs and experiences of the health care providers. Nursing education should embrace the move towards family centred care.

## Conclusion

In conclusion, this study has highlighted the needs of families accompanying injured relatives into the emergency department. These needs were not the same as those identified in the critical care setting and therefore the unique role of the emergency nurse is yet to be defined. Identified issues of concern regarding the care of families include poor communication and discrimination based on nationality. This study provides a platform for the development of holistic trauma and emergency nursing in South Africa. Needs expressed by families should be considered in defining the roles and responsibilities of nurses in practice as well as in the education of nurses.
